# UBE2E1 Is Preferentially Expressed in the Cytoplasm of Slow-Twitch Fibers and Protects Skeletal Muscles from Exacerbated Atrophy upon Dexamethasone Treatment

**DOI:** 10.3390/cells7110214

**Published:** 2018-11-16

**Authors:** Polge Cécile, Aniort Julien, Armani Andrea, Claustre Agnès, Coudy-Gandilhon Cécile, Tournebize Clara, Deval Christiane, Combaret Lydie, Béchet Daniel, Sandri Marco, Attaix Didier, Taillandier Daniel

**Affiliations:** 1 INRA, UMR 1019, Human Nutrition Unit (UNH), 63122 St Genès Champanelle, France; cecile.polge@inra.fr (P.C.); j.aniort@orange.fr (A.J.); agnes.claustre@inra.fr (C.A.); cecile.coudy-gandilhon@inra.fr (C.-G.C.); clara.tournebize@gmail.com (T.C.); christiane.deval@inra.fr (D.C.); lydie.combaret@inra.fr (C.L.); daniel.bechet@inra.fr (B.D.); didier.attaix@inra.fr (A.D.); 2 Venetian Institute of Molecular Medicine, 35100 Padova, Italy; andre.arma88@gmail.com (A.A.); marco.sandri@unipd.it (S.M.)

**Keywords:** ubiquitin-proteasome system, E2 ubiquitin-conjugating enzymes, E3 ubiquitin ligases, UBE2E1, MuRF1, skeletal muscle, atrophy, actin, myosin heavy chain

## Abstract

Skeletal muscle mass is reduced during many diseases or physiological situations (disuse, aging), which results in decreased strength and increased mortality. Muscle mass is mainly controlled by the ubiquitin-proteasome system (UPS), involving hundreds of ubiquitinating enzymes (E2s and E3s) that target their dedicated substrates for subsequent degradation. We recently demonstrated that MuRF1, an E3 ubiquitin ligase known to bind to sarcomeric proteins (telethonin, α-actin, myosins) during catabolic situations, interacts with 5 different E2 enzymes and that these E2-MuRF1 couples are able to target telethonin, a small sarcomeric protein, for degradation. Amongst the E2s interacting with MuRF1, E2E1 was peculiar as the presence of the substrate was necessary for optimal MuRF1-E2E1 interaction. In this work, we focused on the putative role of E2E1 during skeletal muscle atrophy. We found that E2E1 expression was restricted to type I and type IIA muscle fibers and was not detectable in type IIB fibers. This strongly suggests that E2E1 targets are fiber-specific and may be strongly linked to the contractile and metabolic properties of the skeletal muscle. However, E2E1 knockdown was not sufficient for preserving the protein content in C2C12 myotubes subjected to a catabolic state (dexamethasone treatment), suggesting that E2E1 is not involved in the development of muscle atrophy. By contrast, E2E1 knockdown aggravated the atrophying process in both catabolic C2C12 myotubes and the Tibialis anterior muscle of mice, suggesting that E2E1 has a protective effect on muscle mass.

## 1. Introduction

Muscle atrophy is highly deleterious for cachexic patients as it alters both the quality of life and the efficiency of treatments [1,2]. The decrease in muscle mass is attributable to an alteration of proteostasis mainly due to a huge increase of protein degradation, which affects the size of muscle fibers rather than decreasing their number [3,4,5]. In addition, skeletal muscle fibers are differentially affected by the atrophying program depending on their contractile and metablic properties, each fiber type being more sensitive to specific atrophying stimuli [4,5,6]. For example, muscle disuse predominantly affects slow type I fibers while sepsis and cancer cachexia induces atrophy on fast-twitch type II fibers. Proteolysis activation is the main cause of muscle atrophy in numerous catabolic human diseases for the rapid degradation of contractile proteins, the ubiquitin-proteasome system (UPS) and autophagy being the main proteolytic systems involved [3,7]. 

The UPS is crucial as it controls the degradation of the bulk of cellular proteins and also represses protein synthesis [3]. The UPS targets the proteins to be degraded by covalently linking a ubiquitin (Ub) chain to the substrates, which enables the recognition and the degradation of the targets by the 26S proteasome. The Ub chain is formed by an enzymatic cascade that activates Ub (E1 activating enzyme), recognizes the substrates (E3 ligases), and catalyzes an isopeptide linkage between the substrate and Ub or between 2 molecules of Ub (E2 ligases and some E3s). In atrophying skeletal muscles, several UPS genes (Ub, 26S Proteasome subunits, E2s, E3s, etc.) are up-regulated in most catabolic situations, which includes two muscle-specific E3 ligases, muscle atrophy F-box (MAFbx or atrogin1) and muscle ring finger-1 (MuRF1) [8,9,10,11].

MuRF1 is the only E3 ligase known to target the myofibrillar proteins but MuRF1 needs the presence of E2 enzymes for catalyzing the Ub chains [10,12,13,14,15,16]. Little is known about E2 enzymes in muscles (for a review see [17]) but we found recently that at least 5 of them are able to bind MuRF1, including E2E1 [15]. 

E2E1 belongs to the class III E2 enzymes, which means it possesses an N-terminal extension besides the UBC fold that characterizes E2 enzymes [18]. E2E1 is a member of the UBE2E sub-family that comprises also E2E2 and E2E3. The three isoforms have a highly conserved UBC domain (92% identity) but variable N-terminal extension (26% identity) [19,20]. The variability of the latter suggests yet unidentified differential roles between the E2E enzymes that may be related to differential substrate interaction, location, or interacting proteins (E3 ligases, co-factors, etc.). For example, E2E1 and E2E2 can promote ISG15 modification on proteins while E2E3 cannot [21]. Similarly, PTEN is targeted for degradation by E2E1 but not by E2E2 and E2E3 [22], which means that each isoform possesses both common and distinct capacities/roles.

Two reports indicated that the N-terminal extension of E2E1 restricts ubiquitin transfer when Ub is covalently bound to the extension but this mechanism depends on the presence of some E3 ligases [23,24]. In these cases, monoubiquitylation predominates when using the full-length E2E1. However, other studies showed a fair activity of E2E1 for building polyUb chains using other E3 ligases and substrates [23,25,26,27,28]. In addition, E2E1 preferentially catalyzes K48 (or K11) chains on chimeric proteins, i.e., the typical chains for targeting substrates for the subsequent degradation by the 26S proteasome [29]. The discrepancies between the different studies may be linked either to the E3 ligases and/or to the substrates used in the assays.

E2E1 is so far described as mostly nuclear in different organs (human proteome Atlas (https://www.proteinatlas.org)) and cells [30]. The nuclear localization is driven by Importin-11 and is strictly dependent on the presence of Ub in the active site of E2E1 [30]. This preferential location is in line with the mono-Ub of histone H2A by E2E1 in cooperation with the E3 ligase complex PRC1 [31]. However, the location of E2E1 has never been addressed in skeletal muscle cells.

In a previous work, we found that among the E2 enzymes able to interact with MuRF1, E2E1 was peculiar in that only the presence of the substrate (telethonin) allowed for the clear interaction between MuRF1 and E2E1, which suggested a peculiar role during the atrophy process [15]. However, the multiple locations of telethonin and MuRF1 within skeletal muscle cells did not allow us to determine the subcellular location of this interaction. In view of the paucity of data regarding E2E1, we first addressed the expression and location of E2E1 in the skeletal muscles of mice and then we determined its potential role in the muscle during catabolic conditions by using knockdown or overexpression approaches in both cultured cells and mice.

## 2. Materials and Methods

### 2.1. Constructs and Materials

Rat MuRF1 and murine UBE2E1 (E2E1) cDNAs were amplified by RT–PCR from either rat soleus muscles or murine C2C12 myotubes mRNA using Superscript II and Platinum Pfx DNA polymerase (Invitrogen, Carlsbad, CA, USA). cDNAs were cloned in the pcDNA3.1 expression vector (Invitrogen) for expression in mammalian cells. MuRF1 and E2E1 shRNA were produced using pLKO.1 clones purchased from Sigma (Supplementary Table S1). Oligos used for miR production (Supplementary Table S2) directed against E2E1 and the pcDNA™6.2-GW/EmGFP-miR vector were obtained from ThermoFisher Scientific. Combinations of at least 2 RNAi vectors were used for knocking down MuRF1 and E2E1.

Immunoblotting and immunohistochemistry (IHC) were performed using anti-flag (F1804), α-actin (HHF-35), PCNA (P8825), MHCI (M8421), and anti-laminin-α1 (L9393) from Sigma, St. Louis, MO, USA, E2E1 (TA309924), from Origene, Rockville, MD, USA, MHCI (clone BAF8), MHCIIa (clone N2), and MHCIIb (clone BFF3) from DSHB (University of Iowa, Iowa City, IA, USA) and anti-caspase 3 (9662) and cleaved caspase 3 (Asp175, 9661) from Cell Signaling Technology, Danvers, MA, USA. 

### 2.2. Cell Culture and Knockdown Experiments

HEK293T cells and C2C12 myoblasts were cultured in Dulbecco’s Modified Eagle Medium and 10% (*v*/*v*) fetal bovine serum (FBS, Gibco, Invitrogen, Paislay, UK) and supplemented with L-glutamine, non-essential amino acids, and gentamycin (Gibco, growth medium, GM). HEK293T cells were plated in 6-well dishes and transfected by the calcium phosphate co-precipitation method [32]. Cells were transfected or co-transfected with plasmid(s) encoding for green fluorescent protein (GFP, i.e., Mock), MuRF1 or E2E1, and were harvested after 48 h of transfection. Cells were lyzed and soluble proteins were obtained as previously described [32]. Two independent experiments were performed.

For C2C12 myoblast differentiation into myotubes, cells were plated onto 24-well plates and grown to near confluence, and then shifted to DMEM containing 2% horse serum (differentiation medium, DM) for 5 days. A catabolic state was induced in myotubes by adding dexamethasone (Dex, 48 h) before harvesting the cells. 

Lysate from MEF cells invalidated for the PERK kinase and treated for 24 h with 20 µM Resveratrol was used as a positive control of apoptosis (a gift from Dr. J. Averous). A total of 20 µg of soluble proteins (25 mM HEPES, pH 7.4, 400 mM NaCl, 1.5 mM MgCl2, 0.2 mM EDTA, NP40 1%, 1X Sigma inhibitor cocktail) were used for immunoblotting against pro-caspase 3 and cleaved caspase 3. 

Knockdown experiments were carried out on HEK293T and C2C12 myotubes grown in multiwell cell culture plates and treated or not treated with Dex (C2C12 only). Myotubes were transfected or not at day 5 of differentiation with shRNAs targeting either MuRF1, UBE2E1 or no known protein sequence (scr-shRNA, negative control) using either a standards calcium phosphate approach for HEK293T cells as already described [32] or a NEPA21 electroporator (SONIDEL) for C2C12 myotubes. Briefly, electroporation was performed as follows. pLKO.1 constructs were diluted at 0.46 µg/µL in OptiMEM (ThermoFisher Scientific, Waltham, MA, USA). At least 2 different shRNAs were mixed for optimal knockdown (>60%), which was tested by qRT-PCR. Another control group was systematically performed where only 1X PBS was present (mock transfection). 

At day 5 of differentiation, C2C12 myotubes were rinsed twice with 1X PBS and then 300 µL of OptiMEM was added in the wells (24-wells plates). For each well, the myotubes were electroporated following the instructions of the manufacturer with the following parameters: 2 poring pulses, 225 V (5 ms, 10% decay, 50 msec interval); 5 transfer pulses, 30 V (50 ms, 40% decay, 50 ms interval). The DNA solution was removed and 500 µL of fresh growth medium deprived of antibiotic and serum was added in the well. C2C12 myotubes were incubated for 1.5 h at 37 °C under 5% CO_2_ and the medium was replaced by the differentiation medium containing or not dexamethasone (50 µM). Briefly, myotubes were washed in 1X phosphate-buffered saline and 300 µL of lysis buffer (5 mM Tris, pH 7.5, 5 mM EDTA, 1 mM phenylmethylsulfonyl fluoride, 10 mM NEM, 1% Triton X-100/anti-proteases (Protease Inhibitor Mixture/Sigma)) was added. Cells were then scraped off the plate and sonicated for 30 s at maximum power using a UP50H sonicator (Hielscher, Teltow, Germany) as previously described [33]. Cell lysates were then centrifuged at 10,000 *g* for 10 min at 4 °C and the supernatant (soluble fraction) was kept at −80 °C until use. The pellets enriched in myofibrillar proteins were resuspended in a homogenization buffer and sonicated to solubilize the proteins. 

### 2.3. Animals and Knockdown Experiments

The experiments were conducted in accordance with the National Research Council Guide for the Care and Use of Laboratory Animals. National authorization to perform animal experiments for this project has been obtained (authorization #9204 for project 2017042115497506). All animals were maintained in a temperature-controlled room (22 ± 1 °C) with a 12:12 h light:dark cycle. Three-months old male C57BL/6 mice were housed for 1 week in a standard environment and then submitted or not to Dex treatment at 1 or 5 mg/kg/day for 5, 9, or 14 days. The length of the Dex-treatment was adapted to the dose for avoiding excessive weight loss of animals for complying with current legislation on animal care. Food intake was monitored in the first series of experiment and animals were pair-fed for avoiding discrepancies due to Dex treatment. At the end of the experiment, animals were killed by cervical dislocation and the skeletal muscles (EDL, gastrocnemius, soleus, and Tibialis anterior) were excised, weighed, frozen in liquid nitrogen and stored at −80 °C until use. Alternatively, skeletal muscles used for immunohistochemistry were frozen in liquid nitrogen-cooled isopentane. In vivo knockdown was performed as previously described by electroporation [34]. Briefly, a target finder and design tool (Invitrogen) was used to identify target regions in the mouse MuRF1 and E2E1 genes amenable to siRNA. The sequences of siRNAs targeting MuRF1 and E2E1 were cloned into the BLOCK-iT Pol II miR RNAi Expression Vector Kit with EmGFP (Invitrogen number K4936-00). The oligos used are shown in Supplementary Table S2. As a negative control, the pcDNA6.2-GW/EmGFP-miR-neg control was used according to manufacturer’s instructions.

### 2.4. Immunohistochemistry

The contractile type and the cross-sectional area of the T. anterior fibers were determined using serial cross-sections labeled with antibodies directed against the different myosin isoforms and co-labeled with anti-laminin-α1. For E2E1 immunohistochemistry, muscle cross-sections were either pre-incubated in 4% paraformaldehyde to preserve nuclear labeling or directly incubated with a rabbit polyclonal antibody directed against UBE2E1 (UBCH6; Origene, TA309924) to preferentially label muscle fibers. Negative controls were performed using either the secondary FITC-labeled antibody alone or no antibody, the sections being observed at an identical exposure time, i.e., 286 ms (Supplementary Figure S1). Images were captured with a high-resolution cooled digital DP-72 camera coupled to a BX-51 microscope (Olympus, Tokyo, Japan) at a resolution of 0.64 μm/pixel. The CSA and contractile type of each fiber were obtained using the Visilog-6.9 software (Noesis) as previously described [35]. 

### 2.5. Protein Extraction

Portions of the Tibialis anterior muscle (≈10 mg) were homogenized using a TissueRuptor^®^ (Kinematica, Littau-Luzern, Switzerland) in 200 µL of lysis buffer (PBS 1X pH 7.4, 5 mM EDTA pH 8.0, 1 mM PMSF, protease inhibitor cocktail (10 µL/mL of lysis buffer) (Sigma, St Louis, MO, USA), NEM 10 mM, Triton X100 1%). The soluble and the myofibrillar-enriched were separated as previously described [16]. Muscle homogenates were centrifuged at 16,000 *g* (4 °C, 10 min) and the supernatants containing the cytoplasmic fraction were aliquoted and frozen at −80 °C until use. The pellets enriched in myofibrillar proteins were resuspended in 150 µL of lysis buffer containing 2% SDS and 5% glycerol using a sonicator (see above) and frozen at −80 °C until use. 

The protein concentration was measured by absorption spectrophotometry (OD 562 nm) using the BCA kit (Pierce, Rockford, IL, USA) with BSA as a standard. 

### 2.6. qRT-PCR

mRNA levels of muscle-specific E3 ligases (MuRF1, MAFbx) were determined by quantitative RT-PCR (see Supplementary Table S3 for oligos used). The reverse transcription of total RNA into DNA was performed using the QuantiTect^®^ Reverse Transcription kit (Qiagen^®^, Venlo, The Netherlands). qPCR was performed using the FastStart DNA Master SYBR Green I kit (Roche, Basel, Switzerland), according to the manufacturer’s instructions using a CFX96 thermocycler (BIORAD, Hercules, CA, USA). Calculations were made using the comparative ∆Ct method with YWHAZ, HPRT1, and 36B4 housekeeping genes. The oligos used are shown in Supplementary Table S3.

### 2.7. Statistical Analysis

Statistical analyses were performed using the XLSTAT software (Addinsoft^®^, Paris, France). The tests were two-sided, with a Type I error set at α = 0.05. Variables are presented as means ± SE and a Student’s *t*-test or a 1-Way ANOVA (analysis of variance) were performed for cultured cell experiments. For in vivo transfection, the assumption of normality was assessed by the Shapiro–Wilk test and homoscedasticity verified by the Fisher-Snedecor test. Comparisons between groups were performed first using a 1-way ANOVA. When statistical significance was reached, it was followed by multiple post-hoc Tukey’s tests. The transfection of the scr-siRNA and the sh-E2E1 were performed in the left and right leg of the same animal respectively. Data analysis was therefore completed by a multivariate analysis in order to specifically distinguish the “UBE2E1 knockdown” effect from the “transfection” and the “leg” effects. We thus performed a 3-way ANOVA including the 3 factors of variability. The Least Square (LS) means of the surface in control and UBE2E1-knocked down legs were computed using this model and comparisons were made using multiple post-hoc Tukey’s tests. The conclusions of the 1-Way and the 3-Way ANOVA were similar.

## 3. Results and Discussion

We recently identified a MuRF1-E2 network that included E2E1 as a MuRF1 partner [15]. Among the 5 E2 enzymes able to interact with MuRF1, E2E1 was peculiar as it interacted with MuRF1 only when telethonin was present, which was attributed to an allosteric mechanism [15]. MuRF1 is a hallmark of the atrophying process in skeletal muscles and this E3 enzyme recognizes several sarcomeric proteins for their subsequent degradation by the 26S proteasome [12,13,14,15,16]. Little is known about E2E1 and besides the expression levels, no data are available for skeletal muscles. For example, E2E1 is described as a nuclear enzyme in fibroblasts [30] but its location in muscle cells in unknown. As MuRF1 and telethonin are located both in the cytosol and the nucleus [36,37,38], our previous work did not give any clue about a putative role of E2E1 for targeting contractile proteins in the cytoplasm. Thus, in this work, the main objectives were (1) to address the E2E1 location in skeletal muscle cells; (2) to elucidate whether the MuRF1-E2E1 couple is able to target the main contractile proteins (α-actin and myosin heavy chain) for their subsequent degradation; and (3) to identify the putative role of E2E1 during skeletal muscle atrophy in vivo. 

### 3.1. E2E1 Is Present in Both the Nuclei and the Cytoplasm of Mouse and Human Muscle Cells

The human proteome Atlas (https://www.proteinatlas.org) is the widest source of information for the known location of E2E1 together with a single study that found an exclusive nuclear location of E2E1 in murine fibroblasts and in mouse embryo [30]. According to the human proteome Atlas database, E2E1 is mostly nuclear in several organs (e.g., testis) but some cytoplasmic location was also confirmed in colon endothelial cells and in the kidney.

**Figure 1:**
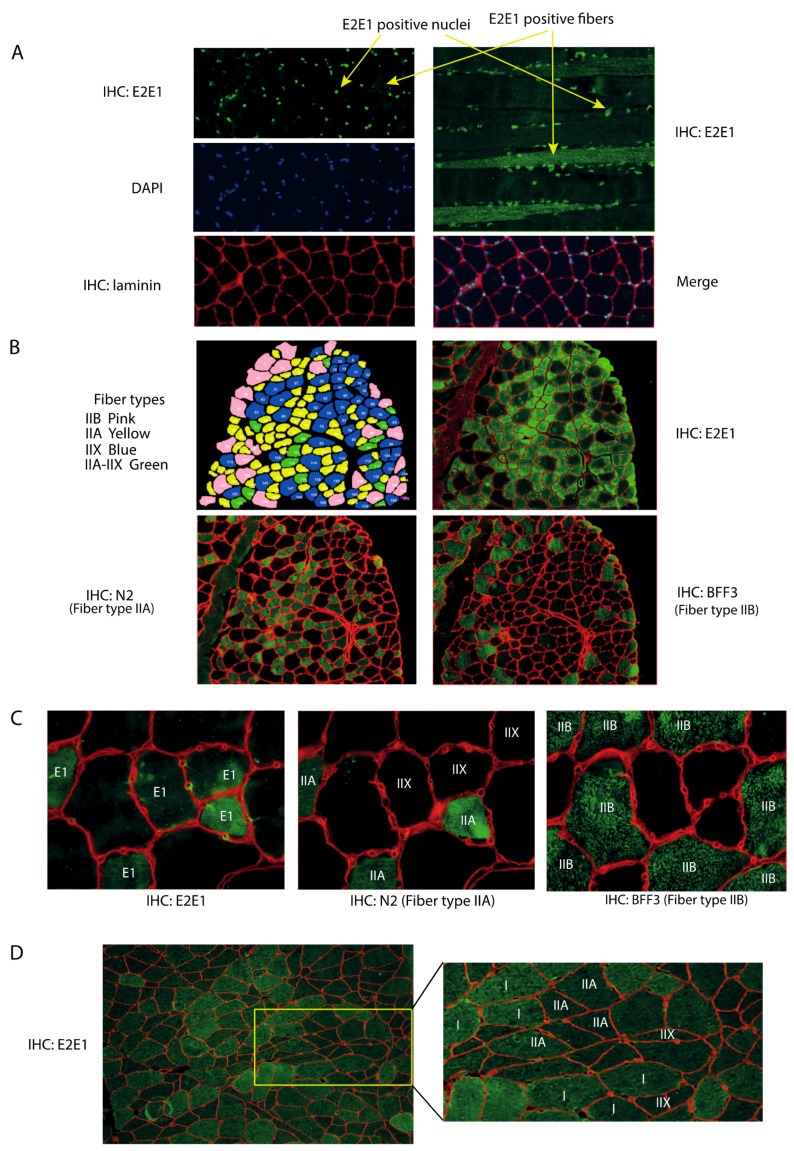
The E2E1 cytoplasmic expression is correlated to the metabolic and contractile properties of fibers. Serial cross-sections of the T. anterior muscle were processed as described in the Methods section. (**A**) Left panels exhibit E2E1 (Top panel), nuclei (DAPI, middle panel) and fiber boundaries (laminin-α1, bottom panel); merged images are shown in the left lower panel. Right upper panel, longitudinal section of T. anterior muscle allowed the detection E2E1 in some fibers; (**B**) Specific identification of fiber types and E2E1 in serial cross-sections showed that E2E1 was predominantly present in type IIA fibers although a faint labeling was also present in type IIX fibers. Results are summarized in false colors in the top left panel; (**C**) Enlargement of a portion of panel B confirms that E2E1 is predominantly expressed in type IIA fibers; (**D**) Human muscle serial cross-sections were used for detecting E2E1 and fiber types. Only E2E1 is shown but the processing was the same as for panel B and C. Only a limited number of fibers are labeled in the enlarged portion of the section (right panel) for more clarity. E2E1 is highly expressed in type I fibers indicating an expression dependent on the metabolic and contractile properties of the cells.

We attempted to locate E2E1 within skeletal muscles for appreciating its potential involvement in the degradation of the contractile apparatus. Indeed, an E2 enzyme exclusively located in the nucleus could not be directly involved in the degradation of contractile proteins in collaboration with MuRF1. Using an immunohistochemistry approach, we observed that E2E1 decorated the nuclei of the mice Tibialis anterior (T. anterior) muscle (Figure 1A), like in other cell types [30]. Interestingly, merging DAPI and E2E1 immunostaining indicated that most if not all the nuclei were positive, indicating that E2E1 was present in the nuclei of any type of fiber (Figure 1A, left panel). Previous work has shown that E2E1 is localized in the nuclei of murine fibroblasts and HeLa cells through an active transport by importin-11 [30]. Interestingly, the nuclear transport of E2E1 is tightly linked to the presence of Ub in the catalytic site of the E2 enzyme and the authors hypothesized that this may be a mechanism for either directly providing the delivery of the activated E2E1 to its nuclear targets or for protecting cytoplasmic targets of E2E1. Recent work confirmed that both hypotheses might be true as the tumor suppressor PTEN was degraded through E2E1 targeting (with the E3 ligase Nedd4) when trapped in the cytoplasm with E2E1, whereas the nuclear import of Ub-E2E1 protected PTEN from degradation [22]. Interestingly, this mechanism was specific of E2E1 as the isoforms E2E2 and E2E3 were unable to ubiquitinylate PTEN. Another recent study found that nuclear E2E1 was part of the PRC1 ligase complex responsible for histone H2A mono-ubiquitylation, which, in turn, repressed the transcription of specific genes [31]. The role of E2E1 in the nuclei of muscle cells is of obvious interest and should be addressed in future work but it was clearly beyond the goals of this study. 

Interestingly, we found a faint although detectable cytoplasmic labeling of E2E1 in the T. anterior cross-sections (Figure 1A, left upper panel). However, longitudinal sections clearly identified some fibers positive for E2E1 staining in the cytoplasm, including the myofibrillar region (Figure 1A, right upper panel). By contrast, no signal was observed in the negative controls using either the secondary FITC-labeled antibody or no antibody (Supplementary Figure S1). E2E1 was uniformly present in the positive fibers and hardly detectable in the negative ones but we cannot rule out that low levels of E2E1 may be present in these fibers. Indeed we did not use long exposure times (≥1 s) because this induces fiber auto-fluorescence due to the presence of myoglobin. As discussed above, the presence/absence of E2E1 in the cytoplasm might be controlled by differential nuclear import fluxes and/or by the modulation of E2E1 expression between fibers. The presence of E2E1 in the cytoplasm of some fibers may be an adaptive mechanism for targeting specific proteins for their degradation.

Within a skeletal muscle, cells do not harbor an identical proteome but rather exhibit different metabolic and contractile properties. This results in a patchwork of cells expressing different enzyme isoforms, more particularly myosin heavy chain (MHC) isoforms [39]. We thus hypothesized that the presence/absence of E2E1 in the cytosol of muscle fibers might be related to the metabolic and contractile properties of the cells, i.e., fiber type, and we sought to verify this hypothesis. First, to better visualize the labeling of E2E1 in the cytoplasm, we eliminated the nuclei of muscle cross-sections (cf. “material and methods” section). Second, using antibodies specific of each MHC (see “material and methods” section), we identified each fiber type. Due to the absence of a specific antibody, note that type IIX fibers were identified by their lack of reactivity when using anti-MHCIIa and MHCIIb antibodies. In other words, they were negative to any of the MHC antibody we used. Using serial cross-sections, we found that cytoplasmic E2E1 was restricted to the intermediate type IIA fibers (and to a lesser extent to type IIX) and was not detectable in fast-twitch type IIB fibers (Figure 1B,C). This reflected an expression directly correlated to the contractile and metabolic properties of muscle fibers. The T. anterior is devoid of type I fibers (slow-twitch fibers), we thus used human biopsies both for confirming the fiber specificity of E2E1 in another organism and for identifying the potential presence of E2E1 in type I fibers. We confirmed that E2E1 is expressed in human type IIA fibers (Figure 1D) and is faintly present in type IIX. It should be noticed that type IIB fibers are absent in humans like in all the muscles from large mammals. Interestingly, we found that E2E1 was also strongly expressed in the cytoplasm of type I fibers, indicating that the E2E1 cytoplasmic location is restricted to slow and intermediate fibers. Although the expression levels were only qualitative, the combined mouse and human data suggest a gradual expression of E2E1 within each fiber type, with I > IIA >> IIX >>> IIB. The simplest hypothesis is that the E2E1 substrates are present in the cytoplasm of slow-twitch and intermediate fibers, which may confer to E2E1 a specific role in skeletal muscle cells expressing a peculiar set of proteins, e.g., myosin heavy chains (MHCI or MHCIIa). As MuRF1 targets different sarcomeric proteins including MHCs, we thus decided to explore whether known substrates of MuRF1 that are expressed either in any fiber type (α-actin [16]) or in specific muscle cells (MHCs [13]) could be potential targets of E2E1.

### 3.2. The MuRF1-E2E1 Couple Is Able to Target α-Actin But Not MHCIIa for Degradation in HEK293T Cells

**Figure 2:**
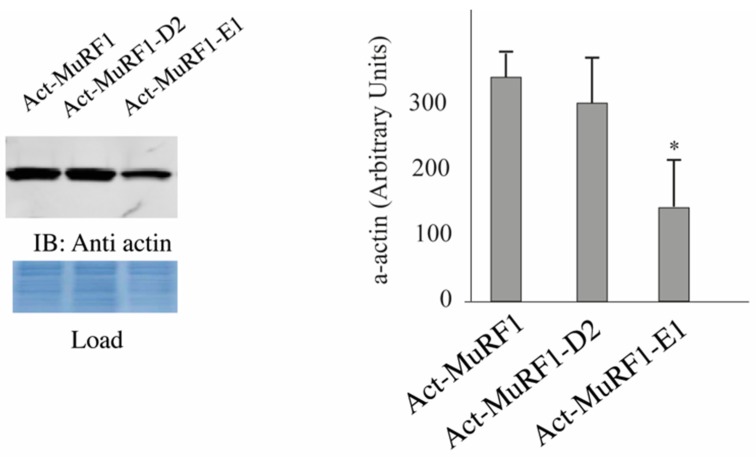
α-actin is targeted for degradation by the MuRF1-E2E1 couple in heterologous cells. Following transfection, α-actin (Act) was expressed in HEK293T cells either alone or with MuRF1 and E2 enzymes; Act-MuRF1, co-transfection of α-actin and MuRF1; Act-MuRF1-D2, co-transfection of α-actin, MuRF1 and E2D2; Act-MuRF1-E1, co-transfection of α-actin, MuRF1, and E2E1. Cells were lyzed and immunoblotting (IB) against α-actin and densitometric analyses were performed as previously described (14). *n* = 6 per group. * Statistically different when compared to Act-MuRF1 group, *p* < 0.05.

**Figure 3:**
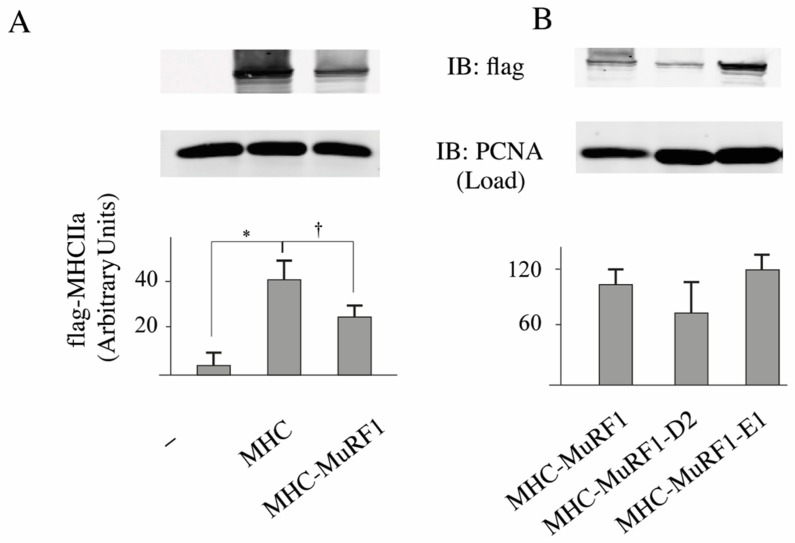
Myosin heavy chain (MHCIIa) is not targeted for degradation by the MuRF1-E2E1 couple in heterologous cells. MHCIIa-flag (MHC) was transfected in HEK293T cells either alone or co-transfected with MuRF1 and different E2 enzymes. Cells were lyzed and immunoblotting (IB) against the flag peptide and densitometric analysis were performed as previously described [16] except that PCNA was used as a loading control. (**A**) We first verified that MHC was expressed in HEK293T cells and that the signal was specific of MHCIIa transfection. In addition, MuRF1 was able to drive part of the MHCIIa protein for degradation in combination with endogenous E2 enzymes; (**B**) E2E1 was not able to promote MHCIIa for degradation in presence of MuRF1. MHC-MuRF1, co-transfection of MHCIIa and MuRF1; MHC-MuRF1-D2, co-transfection of MHCIIa, MuRF1, and E2D2; MHC-MuRF1-E1, co-transfection of MHCIIa, MuRF1 and E2E1. Values are means ± SE for *n* = 6 per group. * Statistically different from controls; † Statistically different from the MHC group, *p* < 0.05.

Due to the high abundance of α-actin and MHCs in muscle cells, detecting modifications of these proteins may be pretty tricky and we thus decided to start our investigations by using heterologous cells that lack α-actin [32] and MHCIIa. We co-transfected HEK293T cells with plasmids encoding for α-actin or MHCIIa and MuRF1 with or without E2E1. As a negative control, we used E2D2 that is not able to catalyze α-actin degradation [32]. We already used this model and demonstrated that α-actin is partially degraded in the presence of MuRF1 thanks to endogenous E2 enzymes present in HEK293T cells [32]. We observed a 58% depression of α-actin levels when E2E1 was co-expressed with MuRF1, while no effect was detected in the presence of E2D2 (Figure 2). We used a similar approach for MHCIIa and first verified that MuRF1 alone was able to target MHCIIa for degradation (−36%, *p* < 0.01; Figure 3A). However, we were not able to detect MHCIIa degradation when E2E1 was over expressed with MuRF1 in HEK293T cells (Figure 3B). Altogether, these data were surprising as E2E1 was able to target a protein present in any type of muscle fiber (α-actin) but not a protein specific of type IIA fibers (MHCIIa), which did not match with the fiber type specificity we observed for E2E1. However, the lack of effect of E2E1 on MHCIIa may be explained by 2 hypotheses: (1) E2E1 is not the E2 enzyme implicated in MHCIIa degradation; (2) E2E1 is lacking a co-factor not present in heterologous HEK293T cells. As a corollary of the latter hypothesis, the co-factor may be present only in atrophying muscle cells. Thus, we moved to a model of cultured skeletal muscle cells (C2C12 myotubes) that can be submitted to catabolic stimuli.

### 3.3. E2E1 Repression Decreased α-Actin Levels and Tended to Depress MHCI Levels in Catabolic C2C12 Myotubes

**Figure 4:**
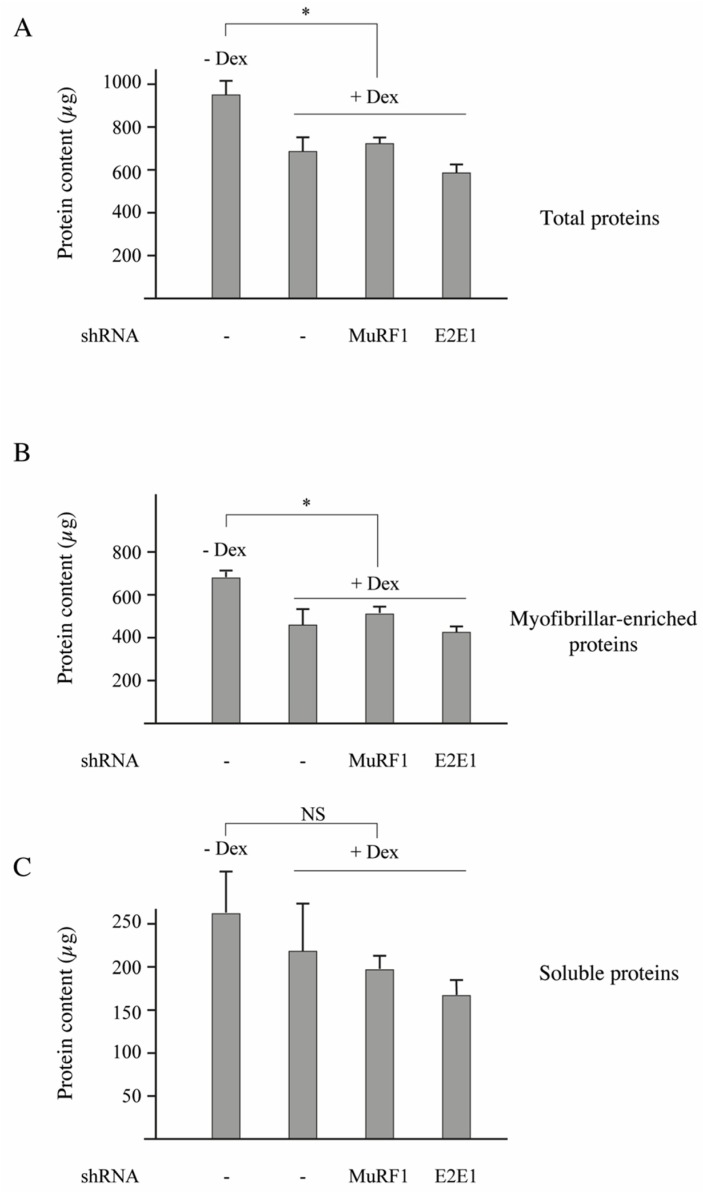
E2E1 knockdown did not significantly modify the overall protein content in catabolic C2C12 myotubes. C2C12 myoblasts were cultured in 24-well plates and differentiated into myotubes for 5 days (d5) as previously described [14]. Using a NEPA21 electroporator, myotubes were transfected at d5 with the shRNAs targeting the indicated E2 and E3 enzymes. C2C12 myotubes were also treated or not with dexamethasone (Dex, 1 µM) at d5. Myotubes were lyzed after 48 h Dex and shRNA treatments and the soluble and myofibrillar fractions were carefully determined as previously described [16]. For more accuracy, we pooled the cell lysates from 4 wells and repeated it 6 times (*n* = 6, but with 24 wells). (**A**) Total proteins; (**B**) Myofibrillar-enriched fraction; (**C**) Soluble fraction. Values are means ± SE for *n* = 6 per group. *, Statistically different from the -Dex group, *p* < 0.01.

C2C12 myotubes were treated or not treated with dexamethasone (Dex, 1 µM) for inducing a catabolic situation. Dex-treatment induced a significant decrease of the total protein content (−33%, *p* < 0,05; Figure 4A) and transfection with shRNAs directed against either MuRF1 or E2E1 did not further modify protein levels (Figure 4A). The total protein decrease was due to a depression of the myofibrillar-enriched fraction with no significant modification of the soluble fraction (Figure 4B,C). 

**Figure 5:**
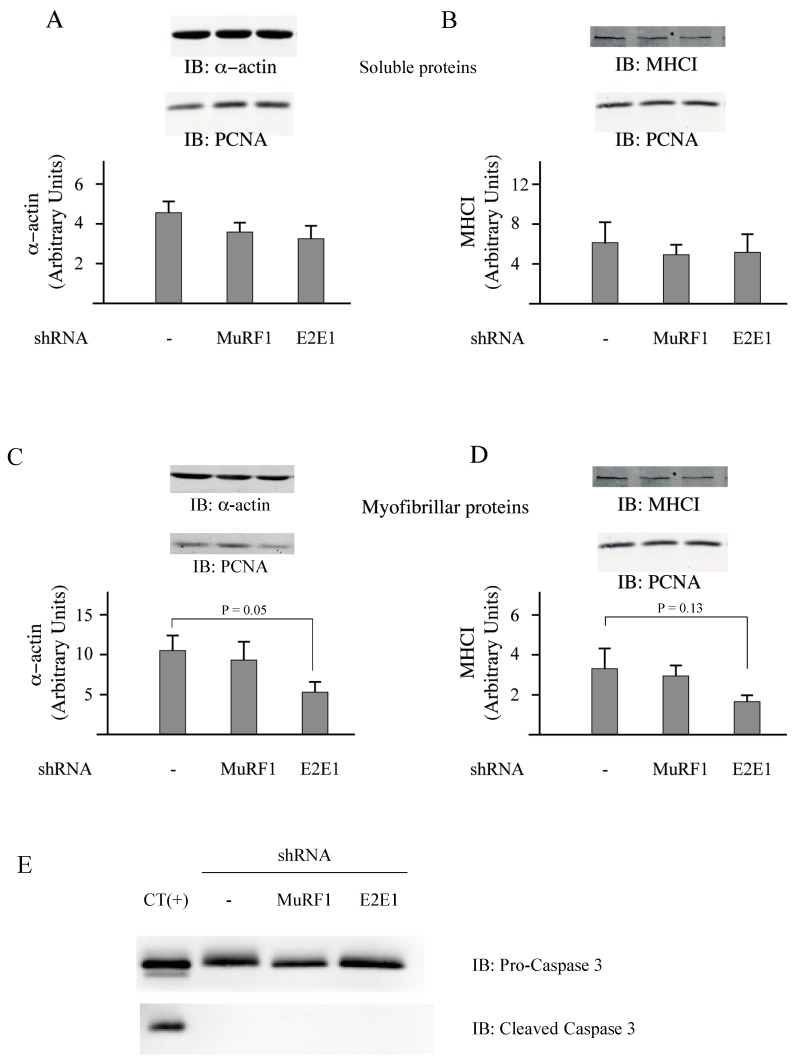
The E2E1 knockdown lowered the myofibrillar α-actin and MHC levels in catabolic C2C12 myotubes. C2C12 myotubes were cultured in 24-well plates, treated with Dex and electroporated as explained in the Figure 4 legend. Total lysate was fractionated in myofibrillar-enriched and soluble proteins as previously described [16]. Soluble (**A**,**B**) and myofibrillar (**B**,**D**) proteins were assayed for α-actin (**A**,**C**) and MHC (**B**,**D**) levels by immunoblotting. PCNA was used as a loading control. Values are means ± SE for *n* = 6 per group. E2E1 knockdown tended to depress α-actin (*p* = 0.05) and MHCIIa (*p* = 0.13) levels in the myofibrillar fraction. (**E**) The levels of Pro-caspase 3 and cleaved caspase 3 (i.e., the active form) were addressed in the soluble fraction (20 µg per lane). As a positive control of the antibodies used, we loaded apoptotic MEF cells lysate (a gift from Dr. J. Averous) (CT(+)). A strong induction of apoptosis (witnessed by caspase 3 cleavage) was detected in these cells. In contrast, we did not detect any activation of caspase 3 in E2E1-knocked down C2C12 myotubes.

Previous work showed that Dex decreased the C2C12 myotube’s diameter (a hallmark of atrophy) by increasing protein degradation, notably through increased levels of MuRF1 and MAFbx [40]. The lack of effect of the shRNAs directed against MuRF1 and E2E1 was not surprising for 2 reasons: (1) contractile proteins represent “only” around 10% of the total proteins in C2C12 myotubes (unpublished data); and (2) in our conditions, we just analyzed a fraction enriched in myofibrillar proteins (total minus soluble proteins). We then addressed the impact of E2E1 knockdown on individual sarcomeric proteins in Dex-treated C2C12 myotubes and MuRF1 knockdown was used as a control. α-actin or MHCI levels were not significantly impacted by the MuRF1 knockdown even though these proteins are MuRF1 targets (Figure 5A–D). This is in accordance with previous studies indicating that the MuRF1 targeting of α-actin can be visualized only in peculiar conditions (e.g., use of chimeric α-actin) because of α-actin abundance [16] and that MHCI targeting depends on both MuRF1 and MuRF3 due to at least a partial redundancy of these E3 ligases [13]. We found that the E2E1 knockdown did not impact α-actin and MHCI levels in the soluble fraction (Figure 5A,B). However, the E2E1 knockdown tended to depress α-actin (−47%, *p* = 0.05) and MHCI content (−50%, *p* = 0.13) in the myofibrillar-enriched fraction (Figure 5C,D). Altogether, the MuRF1-E2E1 couple was able to target α-actin for the degradation in heterologous HEK293T cells but E2E1 was clearly not involved in the enhanced degradation of myofibrillar proteins in catabolic C2C12 myotubes. By contrast, the E2E1 knockdown was even deleterious as it depressed contractile protein levels. 

The knockdown of E2E1 might have indirectly impacted C2C12 myotubes by promoting apoptosis. Indeed, during apoptotic situations (e.g., staurosporine treatment), actin (both the α and the β type) is cleaved by caspase 3 and the fragments are further degraded by the UPS [41,42]. We thus addressed a potential increase of caspase 3 levels and more particularly the cleaved caspase 3, which is the active form. We used PERK^−/−^ MEF cells treated with resveratrol as a positive control of caspase 3 induction [43]. In these cells, a substantial amount of cleaved-caspase 3 (the active form) was detected (Figure 5E, CT(+)), indicating an activation of apoptosis. By contrast, no activated caspase 3 was detected in C2C12 myotubes and the levels of total caspase 3 were similar in control, MuRF1 and E2E1 knocked down cells (Figure 5E). This suggests that the modest reduction in myofibrillar protein levels observed upon E2E1 knockdown was not due to exacerbated apoptosis.

The protection of α-actin and potentially some MHC isoforms by E2E1 may be either direct or indirect and it remains to be determined whether the impact of E2E1 on contractile proteins is due to the manipulation of protein synthesis or degradation. Indeed, the capacity of E2E1 to modulate gene expression in HeLa cells through histone ubiquitylation makes possible an indirect effect of E2E1 on myofibrillar proteins [31]. To our knowledge, no study has addressed the potential control of contractile proteins through histone ubiquitylation and, in this work, we could not attribute the protective effect of E2E1 on catabolic muscle cells to either the nuclear or the cytoplasmic pool of E2E1. This point should be addressed in future investigations but another difficulty resides in the ability of E2E1 to translocate from the cytoplasm to the nucleus, which complicates data interpretation. Indeed, E2E1 may regulate its targets mainly through re-localization rather than *de novo* transcription or translation. The use of E2E1 mutants unable to translocate may help to identify the mechanisms implicated in contractile protein preservation. 

Nevertheless, the data obtained in C2C12 myotubes were quite surprising as they suggested that the presence of E2E1 was needed for lowering the effects of Dex and thus protecting the contractile apparatus during a catabolic situation. This was in contradiction with the capacity of E2E1 to drive α-actin degradation in heterologous cells (see Figure 2). However, the data obtained in HEK293T cells may not be representative of the E2E1 role in muscle cells or may only represent one of its multiple roles. For clarifying these discrepant results, we decided to address the impact of E2E1 in catabolic skeletal muscles from mice treated with Dex.

### 3.4. Dex-Treatment Induces Muscle Atrophy and Activates the UPS in Mice

Dex treatment induces muscle atrophy in humans and rodents [16,44,45]. However, the concentration and the timing of Dex that induce both muscle atrophy and increased activation of proteolytic systems in mice greatly varies in the literature from 1 to 25 mg/kg/day with different injection modes (intraperitoneally, gavage, etc.) [44,46,47]. In addition, few studies addressed both muscle atrophy and the activation of the UPS. Furthermore, corticoids like Dex may alter food intake in mice by increasing the appetite [48,49], which may modify proteostasis. Thus, we first set up the best conditions that induce skeletal muscle atrophy and activate the UPS, with a special focus on the E3 ligase involved in contractile protein targeting, i.e., MuRF1.

**Figure 6:**
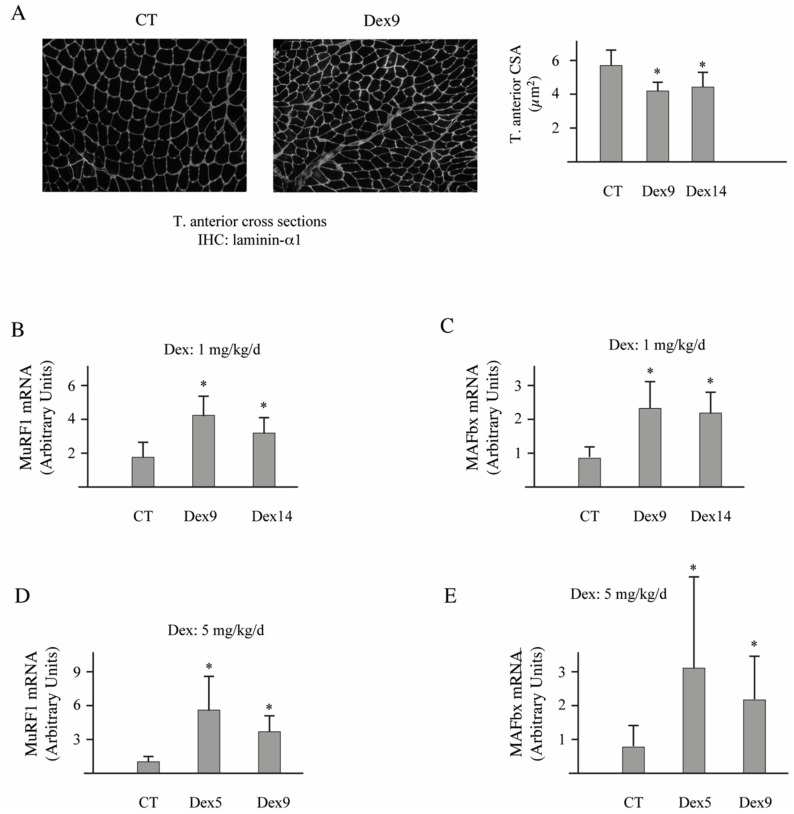
The Dex-treatment induced muscle atrophy in C57BL/6 mice. Animals were treated or not treated with Dex at 1 or 5 mg/kg/d for 5 to 14 days. The length of Dex-treatment was adapted to the dose for avoiding excessive weight loss of animals. (**A**) IHC was performed using anti-laminin-α1 (left and middle panels) and the fiber cross-sectional area was determined using the Visilog-6.9 software (right panel). The lowest dose of Dex was efficient for inducing muscle atrophy and depressing the fiber diameter by −22 to −24%; (**B**,**C**) The expression levels of the E3 ligases MuRF1; (**C**) and MAFbx (**D**) were addressed by qRT-PCR in mice subjected to Dex-treatment (1 mg/kg/d). More than a 2-fold increase was observed for both E3 ligases; (**D**,**E**) Same as (**B**,**C**) but with 5 mg/kg/d of Dex. A 3 to 5-fold increase of both MuRF1 and MAFbx mRNA levels was observed. Values are means ± SE for *n* = 4–5 per group. *, Significantly different from control (CT) group, *p* < 0.05.

Mice food intake was individually monitored for 1 week before initiating the experiment. Thereafter, Dex-treated animals were pair-fed according to control levels. However, we did not notice any detectable variation of food intake (not shown), which indicated that nutrient availability could not interfere with the Dex-treatment. We tested two doses of Dex (1 and 5 mg/d/kg) dissolved in drinking water for 5, 9, or 14 days. Both doses of Dex were efficient whatever the time of experiment and induced a significant muscle atrophy that paralleled the total body weight loss of animals (Table 1). However, atrophy was slightly higher for the four muscles tested (gastrocnemius, soleus, T. anterior, and EDL) for 5-days of treatment at 5 mg/kg/d. Accordingly, muscle weight loss was accompanied by decreased cross-sectional areas in the T. anterior muscle (Figure 6A, −22/−24%, *p* < 0.05). 

The two E3 ligases MuRF1 and MAFbx are considered as the best markers of muscle atrophy even in moderate atrophying conditions [10,50]. We observed that both doses of Dex efficiently up-regulated MuRF1 and MAFbx in the T. anterior muscle for any of the time points tested (Figure 6B–E). However, MuRF1 mRNA levels increased more with the dose of 5 mg/kg/d (5-days of treatment, +560%, *p* < 0.01; 9-days of treatment, +360%, *p* < 0.01) than with 1 mg/kg/d (9-days of treatment, +246%, *p* < 0.01; 14-days of treatment, +85%, *p* < 0.01). As Dex treatment at 5 mg/kg/d for 5 days induced higher levels of atrophy and MuRF1 up regulation, we used this condition for addressing the potential role of E2E1 in vivo in skeletal muscles.

### 3.5. E2E1 Knockdown Aggravates Muscle Atrophy in Dex-Treated Mice

**Figure 7:**
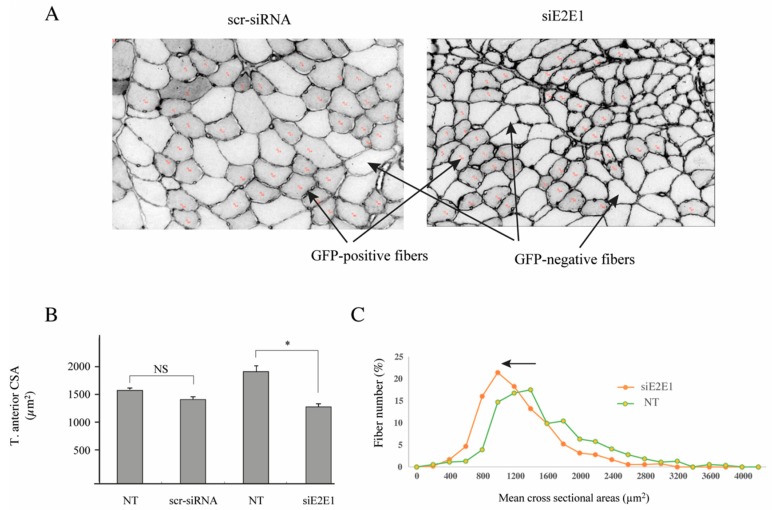
The E2E1 knockdown induced a decrease of cross-sectional area (CSA). Mice were transfected either with a scramble siRNA (scr-siRNA, left leg) targeting no known coding sequence or with a mixture of 4 siRNAs (siE2E1, right leg) directed against E2E1. Each plasmid also encoded for emGFP, which allowed for identifying transfected and non-transfected cells within each muscle. Cross-sections were also labeled with anti-laminin-α1 for determining CSA (see Methods section and Figure 6 legend for details). Two different zones and a minimum of 100 fibers were used for each muscle. (**A**) GFP positive fibers were visually smaller in siE2E1-treated fibers; a typical result is shown; (**B**) CSA analysis from *n* = 6 mice and a total of 1449 fibers were analyzed using a 1-way ANOVA and confirmed using a 3-way ANOVA. A 31% decrease of CSA was observed upon E2E1 knockdown (siE2E1) in GFP-positive fibers (i.e., transfected fibers, siE2E1 group) when compared to GFP-negative fibers (i.e., non-transfected fibers, NT group). There was no difference when muscles were transfected with the scramble siRNA. *, Statistically different from the NT group, *p* < 0.001); (**C**) Fibers distribution was analyzed within each muscle by grouping CSAs by 200 µm^2^ steps. A global shift towards lower CSAs was observed in the E2E1 knocked down fibers when compared to their corresponding controls (NT).

Another series of mice were transfected with scramble siRNA (scr-siRNA, negative control) or with a mixture of siRNA directed against E2E1 (siE2E1, knockdown). The same animal was used for both transfections, with the left leg being the control (scr-siRNA) and the right leg being the knocked down (siE2E1). After 4 days, the mice were treated with Dex (5 mg/d/kg) for 5 days. The plasmids encoding for the siRNAs also expressed emGFP, which allowed identifying the transfected cells. The average efficiency of cell transfection was 55 and 56% in scr-siRNA and siE2E1 transfected muscles respectively and 100–160 fibers per leg were analyzed (total fibers, 1449). We took advantage of the presence of both transfected (GFP-positive) and non-transfected (GFP-negative) cells within the same muscle to compare the fiber size between the 2 populations. We found that the scr-siRNA did not affect the fiber cross-sectional area (Figure 7A left panel and Figure 7B). By contrast, the knockdown of E2E1 induced a global decrease in cell size (−32%, *p* < 0.001, Figure 7A right panel and Figure 7B), which showed that the E2E1 knockdown negatively impacted the catabolic T. anterior muscle. The decrease in fiber size homogenously impacted the muscle as indicated by the global shift of fiber cross-sectional area towards lower sizes (Figure 7C). As cytoplasmic E2E1 is present in type IIA fibers, the latter may be more sensitive to E2E1 knockdown. Thus, we further analyzed whether the E2E1 knockdown may have impacted differentially the fibers depending on their metabolic and contractile properties. No significant difference was detected, indicating that the decrease of fiber size affected all the fibers (not shown). In addition, E2E1 knockdown did not modify α-actin and MHCIIa levels (Supplementary Figure S2), indicating that muscle atrophy was homogeneous.

Based on the effect of MuRF1-E2E1 on telethonin, we first hypothesized that these ubiquitinating enzymes could be responsible for the degradation of contractile proteins in catabolic skeletal muscles. However, like MuRF1, telethonin is present not only in the contractile apparatus where it interacts with the giant protein titin but also as a free protein either in the cytoplasm or in the nucleus [38,51]. Indeed, telethonin possesses pleiotropic functions including regulatory ones and the MuRF1-dependent degradation of telethonin may not be related to the sarcomeric function of telethonin. We show in this work that E2E1 is both cytosolic and nuclear and that the main in vivo function of E2E1 is not to promote muscle cell atrophy. In contrast, E2E1 is necessary for avoiding excessive muscle loss in Dex-treated mice. The dual location of E2E1 suggests different functions with probably different E3 ligases and future investigations will have to identify which pool of E2E1 is crucial for avoiding excessive muscle atrophy. The importance of E2E1 for maintaining skeletal muscle cell size is in accordance with the deleterious effect of E2E1 knockdown observed in cultured C2C12 myotubes, i.e., depressed levels of α-actin and a tendency to reduce MHC content (see Figure 5). 

An interesting question is whether the 2 other isoforms of the E2E sub-family are also located and/or expressed differentially among the different fiber types. It is nevertheless tempting to hypothesize at least partially divergent roles for the E2E isoforms on the basis of what is already known about E2 enzymes. Indeed, a single amino acid modification is able to change the catalytic activity of an E2 (see Reference [17] for a review). This means that the highly divergent N-terminal extensions may drastically orientate the enzymes to very different fate. This is highlighted by the already known implication of E2E enzymes in diseases, with E2E1 involved in cancer and Sjogren’s syndrome, E2E2 in diabetes and E2E3 in Liddle’s syndrome [52]. 

## 4. Conclusions

Future work will have to determine which E3 ligase is implicated in the protective role of E2E1 during muscle atrophy; indeed, at least twenty E3 ligases are able to interact with E2E1 [53]. A striking result of our work was the selective expression pattern of E2E1 within the different fiber types. The major proteins that characterize the different fibers are myosin heavy chains, but numerous protein isoforms participate in the difference between the slow, intermediate and fast fibers. A major future goal will be to determine whether the specific expression pattern of E2E1 is related to specific protein isoforms and, consequently, to identify them. 

## Figures and Tables

**Table 1:** The effect of dexamethasone treatment on hindlimb muscles. Two independent experiments were conducted for defining the best timing and dexamethasone (Dex) concentration for subsequent experiments.

	Experiment 1			Experiment 2		
	Dex 1 mg/d/kg			Dex 5 mg/d/kg		
	CT	Dex9	Dex14	CT	Dex5	Dex9
**Mice (g)**	26.01 ± 1.8	22.6 ± 1.4 *	21.9 ± 2.2 *	28.1 ± 1.8	24.6 ± 0.4 *	24.2 ± 0.6 *
**Gastrocnemius (mg)**	133.5 ± 8.0	112.0 ± 2.4 *	94.9 ± 10.3 *	163.7 ± 8.6	117.2 ± 5.4 *	118.4 ± 7.5 *
**T. anterior (mg)**	42.9 ± 2.0	39.1 ± 0.7 *	33.94 ± 3.8 *	51.5 ± 1.5	40.2 ± 1.7 *	43.7 ± 2.4 *
**EDL (mg)**	9.9 ± 0.9	8.4 ± 0.4 *	7.08 ± 1.0 *	12 ± 0.7	8.8 ± 0.5 *	9.1 ± 0.4 *
**Soleus (mg)**	8.5 ± 1.5	7.4 ± 0.6	7.3 ± 0.6	9.3 ± 0.7	7.6 ± 0.3 *	7.9 ± 0.6 *

**Experiment 1**: Twelve-week male C57Bl6 mice were housed in standard conditions with free access to water. Food consumption was monitored for 1 week before submitting mice to (Dex) treatment. At day 0, mice were randomly divided into the control (CT), 9-days of Dex treatment (Dex9), and 14-days of Dex treatment (Dex14) and treated with Dex. Dex was solubilized in drinking water and the desired Dex ingestion (1 mg/d/kg) was calculated according to previously determined water consumption. Dex-treated animals were pair-fed according to CT group as Dex treatment increases food consumption in mice. At the end of the experiment, animals were anesthetized (fluorane) and killed by cervical dislocation. Gastrocnemius, Tibialis anterior, Extensor Digitorum Longus, and Soleus skeletal muscles were excised and weighted. The gastrocnemius and T. anterior muscles from the right leg were frozen in liquid nitrogen while the muscles from the left leg were mounted for subsequent immunohistochemistry and frozen using liquid nitrogen-cooled isopentane. **Experiment 2:** Same as pre-experiment 1 but with a dose of 5 mg/d/kg of Dex and 5 or 9 days of treatment. Note that the animals were bigger in this experiment, hence, having bigger skeletal muscles. Values are means ± SE for *n* = 5 per group. *, Statistically different from the CT group, *p* < 0.05.

## Supplementary Materials

The following are available online at http://www.mdpi.com/2073-4409/7/11/214/s1.

## References

[1] Fearon K., Arends J., Baracos V. (2013). Understanding the mechanisms and treatment options in cancer cachexia. Nature reviews. Clin. Oncol..

[2] Von Haehling S., Anker M.S., Anker S.D. (2016). Prevalence and clinical impact of cachexia in chronic illness in Europe, USA, and Japan: Facts and numbers update 2016. J. Cachexia Sarcopenia Muscle.

[3] Sandri M. (2013). Protein breakdown in muscle wasting: Role of autophagy-lysosome and ubiquitin-proteasome. Int. J. Biochem. Cell Biol..

[4] Wang Y., Pessin J.E. (2013). Mechanisms for fiber-type specificity of skeletal muscle atrophy. Curr. Opin. Clin. Nutr. Metab. Care.

[5] Talbot J., Maves L. (2016). Skeletal muscle fiber type: Using insights from muscle developmental biology to dissect targets for susceptibility and resistance to muscle disease. Wiley interdisciplinary reviews. Dev. Biol..

[6] Ciciliot S., Rossi A.C., Dyar K.A., Blaauw B., Schiaffino S. (2013). Muscle type and fiber type specificity in muscle wasting. Int. J. Biochem. Cell Biol..

[7] Milan G., Romanello V., Pescatore F., Armani A., Paik J.H., Frasson L., Seydel A., Zhao J., Abraham R., Goldberg A.L. (2015). Regulation of autophagy and the ubiquitin-proteasome system by the FoxO transcriptional network during muscle atrophy. Nat. Commun..

[8] Attaix D., Ventadour S., Codran A., Béchet D., Taillandier D., Combaret L. (2005). The ubiquitin-proteasome system and skeletal muscle wasting. Essays Biochem..

[9] Lecker S.H., Jagoe R.T., Gilbert A., Gomes M., Baracos V., Bailey J., Price S.R., Mitch W.E., Goldberg A.L. (2004). Multiple types of skeletal muscle atrophy involve a common program of changes in gene expression. FASEB J..

[10] Bodine S.C., Baehr L.M. (2014). Skeletal muscle atrophy and the E3 ubiquitin ligases MuRF1 and MAFbx/atrogin-1. Am. J. Physiol. Endocrinol. Metab..

[11] Bodine S.C., Latres E., Baumhueter S., Lai V.K., Nunez L., Clarke B.A., Poueymirou W.T., Panaro F.J., Na E., Dharmarajan K. (2001). Identification of ubiquitin ligases required for skeletal muscle atrophy. Science.

[12] Clarke B.A., Drujan D., Willis M.S., Murphy L.O., Corpina R.A., Burova E., Rakhilin S.V., Stitt T.N., Patterson C., Latres E. (2007). The E3 Ligase MuRF1 degrades myosin heavy chain protein in dexamethasone-treated skeletal muscle. Cell Metab..

[13] Fielitz J., Kim M.-S., Shelton J.M., Latif S., Spencer J.A., Glass D.J., Richardson J.A., Bassel-Duby R., Olson E.N. (2007). Myosin accumulation and striated muscle myopathy result from the loss of muscle RING finger 1 and 3. J. Clin. Investig..

[14] Kedar V., McDonough H., Arya R., Li H.H., Rockman H.A., Patterson C. (2004). Muscle-specific ring finger 1 is a bona fide ubiquitin ligase that degrades cardiac troponin I. Proc. Natl. Acad. Sci. USA.

[15] Polge C., Cabantous S., Deval C., Claustre A., Hauvette A., Bouchenot C., Aniort J., Béchet D., Combaret L., Attaix D. (2018). A muscle-specific MuRF1-E2 network requires stabilization of MuRF1-E2 complexes by telethonin, a newly identified substrate. J. Cachexia Sarcopenia Muscle.

[16] Polge C., Heng A.-E., Jarzaguet M., Ventadour S., Claustre A., Combaret L., Bechet D., Matondo M., Uttenweiler-Joseph S., Monsarrat B. (2011). Muscle actin is polyubiquitinylated in vitro and in vivo and targeted for breakdown by the E3 ligase MuRF1. FASEB J..

[17] Polge C., Attaix D., Taillandier D. (2015). Role of E2-Ub-conjugating enzymes during skeletal muscle atrophy. Front. Physiol..

[18] Van Wijk S.J., Timmers H.T. (2010). The family of ubiquitin-conjugating enzymes (E2s): Deciding between life and death of proteins. FASEB J..

[19] Markson G., Kiel C., Hyde R., Brown S., Charalabous P., Bremm A., Semple J., Woodsmith J., Duley S., Salehi-Ashtiani K. (2009). Analysis of the human E2 ubiquitin conjugating enzyme protein interaction network. Genome Res..

[20] Schumacher F.R., Wilson G., Day C.L. (2013). The N-terminal extension of UBE2E ubiquitin-conjugating enzymes limits chain assembly. J. Mol. Biol..

[21] Takeuchi T., Iwahara S., Saeki Y., Sasajima H., Yokosawa H. (2005). Link between the ubiquitin conjugation system and the ISG15 conjugation system: ISG15 conjugation to the UbcH6 ubiquitin E2 enzyme. J. Biochem..

[22] Chen M., Nowak D.G., Narula N., Robinson B., Watrud K., Ambrico A., Herzka T.M., Zeeman M.E., Minderer M., Zheng W. (2017). The nuclear transport receptor Importin-11 is a tumor suppressor that maintains PTEN protein. J. Cell Biol..

[23] Nguyen L., Plafker K.S., Starnes A., Cook M., Klevit R.E., Plafker S.M. (2014). The ubiquitin-conjugating enzyme, UbcM2, is restricted to monoubiquitylation by a two-fold mechanism that involves backside residues of E2 and Lys48 of ubiquitin. Biochemistry.

[24] Christensen D.E., Brzovic P.S., Klevit R.E. (2007). E2-BRCA1 RING interactions dictate synthesis of mono- or specific polyubiquitin chain linkages. Nat. Struct. Mol. Biol..

[25] Banka P.A., Behera A.P., Sarkar S., Datta A.B. (2015). RING E3-Catalyzed E2 Self-Ubiquitination Attenuates the Activity of Ube2E Ubiquitin-Conjugating Enzymes. J. Mol. Biol..

[26] Napolitano L.M., Jaffray E.G., Hay R.T., Meroni G. (2011). Functional interactions between ubiquitin E2 enzymes and TRIM proteins. Biochem. J..

[27] David Y., Ziv T., Admon A., Navon A. (2010). The E2 ubiquitin-conjugating enzymes direct polyubiquitination to preferred lysines. J. Biol. Chem..

[28] Sarkari F., Wheaton K., La Delfa A., Mohamed M., Shaikh F., Khatun R., Arrowsmith C.H., Frappier L., Saridakis V., Sheng Y. (2013). Ubiquitin-specific protease 7 is a regulator of ubiquitin-conjugating enzyme UbE2E1. J. Biol. Chem..

[29] David Y., Ternette N., Edelmann M.J., Ziv T., Gayer B., Sertchook R., Dadon Y., Kessler B.M., Navon A. (2011). E3 ligases determine ubiquitination site and conjugate type by enforcing specificity on E2 enzymes. J. Biol. Chem..

[30] Plafker S.M., Plafker K.S., Weissman A.M., Macara I.G. (2004). Ubiquitin charging of human class III ubiquitin-conjugating enzymes triggers their nuclear import. J. Cell Biol..

[31] Wheaton K., Sarkari F., Stanly Johns B., Davarinejad H., Egorova O., Kaustov L., Raught B., Saridakis V., Sheng Y. (2017). UbE2E1/UBCH6 Is a Critical in Vivo E2 for the PRC1-catalyzed Ubiquitination of H2A at Lys-119. J. Biol. Chem..

[32] Polge C., Koulmann N., Claustre A., Jarzaguet M., Serrurier B., Combaret L., Béchet D., Bigard X., Attaix D., Taillandier D. (2016). UBE2D2 is not involved in MuRF1-dependent muscle wasting during hindlimb suspension. Int. J. Biochem. Cell Biol..

[33] Ventadour S., Jarzaguet M., Wing S.S., Chambon C., Combaret L., Bechet D., Attaix D., Taillandier D. (2007). A new method of purification of proteasome substrates reveals polyubiquitination of 20 S proteasome subunits. J. Biol. Chem..

[34] Soares R.J., Cagnin S., Chemello F., Silvestrin M., Musaro A., De Pitta C., Lanfranchi G., Sandri M. (2014). Involvement of microRNAs in the regulation of muscle wasting during catabolic conditions. J. Biol. Chem..

[35] Gueugneau M., Coudy-Gandilhon C., Théron L., Meunier B., Barboiron C., Combaret L., Taillandier D., Polge C., Attaix D., Picard B. (2015). Skeletal muscle lipid content and oxidative activity in relation to muscle fiber type in aging and metabolic syndrome. J. Gerontol. Ser. A Biol. Sci. Med. Sci..

[36] Ochala J., Gustafson A.-M., Diez M.L., Renaud G., Li M., Aare S., Qaisar R., Banduseela V.C., Hedström Y., Tang X. (2011). Preferential skeletal muscle myosin loss in response to mechanical silencing in a novel rat intensive care unit model: Underlying mechanisms. J. Physiol..

[37] McElhinny A.S., Kakinuma K., Sorimachi H., Labeit S., Gregorio C.C. (2002). Muscle-specific RING finger-1 interacts with titin to regulate sarcomeric M-line and thick filament structure and may have nuclear functions via its interaction with glucocorticoid modulatory element binding protein-1. J. Cell Biol..

[38] Knöll R., Linke W.A., Zou P., Miocic S., Kostin S., Buyandelger B., Ku C.H., Neef S., Bug M., Schäfer K. (2011). Telethonin deficiency is associated with maladaptation to biomechanical stress in the mammalian heart. Circ. Res..

[39] Schiaffino S. (2018). Muscle fiber type diversity revealed by anti-myosin heavy chain antibodies. FEBS J..

[40] Menconi M., Gonnella P., Petkova V., Lecker S., Hasselgren P.O. (2008). Dexamethasone and corticosterone induce similar, but not identical, muscle wasting responses in cultured L6 and C2C12 myotubes. J. Cell. Biochem..

[41] Mashima T., Naito M., Tsuruo T. (1999). Caspase-mediated cleavage of cytoskeletal actin plays a positive role in the process of morphological apoptosis. Oncogene.

[42] Du J., Wang X., Miereles C., Bailey J.L., Debigare R., Zheng S., Price S.R., Mitch W.E. (2004). Activation of caspase-3 is an initial step triggering accelerated muscle proteolysis in catabolic conditions. J. Clin. Investig..

[43] Wu X., Xu Y., Zhu B., Liu Q., Yao Q., Zhao G. (2018). Resveratrol induces apoptosis in SGC-7901 gastric cancer cells. Resveratrol induces apoptosis in SGC-7901 gastric cancer cells. Oncol. Lett..

[44] Umeki D., Ohnuki Y., Mototani Y., Shiozawa K., Suita K., Fujita T., Nakamura Y., Saeki Y., Okumura S. (2015). Protective Effects of Clenbuterol against Dexamethasone-Induced Masseter Muscle Atrophy and Myosin Heavy Chain Transition. PLoS ONE.

[45] Reeves E.K., Rayavarapu S., Damsker J.M., Nagaraju K. (2012). Glucocorticoid analogues: Potential therapeutic alternatives for treating inflammatory muscle diseases. Endocr. Metab. Immune Disord. Drug Targets.

[46] Nakao R., Yamamoto S., Yasumoto Y., Oishi K. (2014). Dosing schedule-dependent attenuation of dexamethasone-induced muscle atrophy in mice. Chronobiol. Int..

[47] Protzek A.O.P., Costa-Júnior J.M., Rezende L.F., Santos G.J., Araújo T.G., Vettorazzi J.F., Ortis F., Carneiro E.M., Rafacho A., Boschero A.C. (2014). Augmented beta-Cell Function and Mass in Glucocorticoid-Treated Rodents Are Associated with Increased Islet Ir-beta/AKT/mTOR and Decreased AMPK/ACC and AS160 Signaling. Int. J. Endocrinol..

[48] Arvaniti K., Ricquier D., Champigny O., Richard D. (1998). Leptin and corticosterone have opposite effects on food intake and the expression of UCP1 mRNA in brown adipose tissue of lep(ob)/lep(ob) mice. Endocrinology.

[49] Gounarides J.S., Korach-André M., Killary K., Argentieri G., Turner O., Laurent D. (2008). Effect of dexamethasone on glucose tolerance and fat metabolism in a diet-induced obesity mouse model. Endocrinology.

[50] Aniort J., Polge C., Claustre A., Combaret L., Béchet D., Attaix D., Heng A.-E., Taillandier D. (2016). Upregulation of MuRF1 and MAFbx participates to muscle wasting upon gentamicin-induced acute kidney injury. Int. J. Biochem. Cell Biol..

[51] Nicholas G., Thomas M., Langley B., Somers W., Patel K., Kemp C.F., Sharma M., Kambadur R. (2002). Titin-cap associates with, and regulates secretion of, Myostatin. J. Cell. Physiol..

[52] Hormaechea-Agulla D., Kim Y., Song M.S., Song S.J. (2018). New Insights into the Role of E2s in the Pathogenesis of Diseases: Lessons Learned from UBE2O. Mol. Cells.

[53] Van Wijk S.J.L., de Vries S.J., Kemmeren P., Huang A., Boelens R., Bonvin A.M.J.J., Timmers H.T.M. (2009). A comprehensive framework of E2-RING E3 interactions of the human ubiquitin-proteasome system. Mol. Syst. Biol..

