# Erratum: Polge, C., et al. UBE2E1 Is Preferentially Expressed in the Cytoplasm of Slow-Twitch Fibers and Protects Skeletal Muscles from Exacerbated Atrophy upon Dexamethasone Treatment. *Cells* 2018, *7*, 214

**DOI:** 10.3390/cells7120242

**Published:** 2018-12-04

**Authors:** Cécile Polge, Julien Aniort, Andrea Armani, Agnès Claustre, Cécile Coudy-Gandilhon, Clara Tournebize, Christiane Deval, Lydie Combaret, Daniel Béchet, Marco Sandri, Didier Attaix, Daniel Taillandier

**Affiliations:** 1 INRA, UMR 1019, Human Nutrition Unit (UNH), 63122 St Genès Champanelle, France; cecile.polge@inra.fr (C.P.); j.aniort@orange.fr (J.A.); agnes.claustre@inra.fr (A.C.); cecile.coudy-gandilhon@inra.fr (C.C.-G.); clara.tournebize@gmail.com (C.T.); christiane.deval@inra.fr (C.D.); lydie.combaret@inra.fr (L.C.); daniel.bechet@inra.fr (D.B.); didier.attaix@inra.fr (D.A.); 2 Venetian Institute of Molecular Medicine, 35100 Padova, Italy; andre.arma88@gmail.com (A.A.); marco.sandri@unipd.it (M.S.)

Change in author names (order).

The authors wish to make the following corrections to this paper [1]:

Cécile Polge ^1^, Julien Aniort ^1^, Andrea Armani ^2^, Agnès Claustre ^1^, Cécile Coudy-Gandilhon ^1^, Clara Tournebize ^1^, Christiane Deval ^1^, Lydie Combaret ^1^, Daniel Béchet ^1^, Marco Sandri ^2^, Didier Attaix ^1^ and Daniel Taillandier ^1,^*

^1^  INRA, UMR 1019, Human Nutrition Unit (UNH), 63122 St Genès Champanelle, France; cecile.polge@inra.fr (C.P.); j.aniort@orange.fr (J.A.); agnes.claustre@inra.fr (A.C.); cecile.coudy-gandilhon@inra.fr (C.C.-G.); clara.tournebize@gmail.com (C.T.); christiane.deval@inra.fr (C.D.); lydie.combaret@inra.fr (L.C.); daniel.bechet@inra.fr (D.B.); didier.attaix@inra.fr (D.A.)
^2^  Venetian Institute of Molecular Medicine, 35100 Padova, Italy; andre.arma88@gmail.com (A.A.); marco.sandri@unipd.it (M.S.)

The authors would like to apologize for any inconvenience caused to the readers by these changes. The changes do not affect the scientific results.
