# Supplementary material for: UBE2E1 Is Preferentially Expressed in the Cytoplasm of Slow-Twitch Fibers and Protects Skeletal Muscles from Exacerbated Atrophy upon Dexamethasone Treatment
Source: Cells. 2018 Nov 16;7(11):214. doi: 10.3390/cells7110214 (PMC6262581; doi:10.3390/cells7110214)
Supplement: Supplementary file 1 [file cells-07-00214-s001.pdf]

cells

Supplemental Figure 1

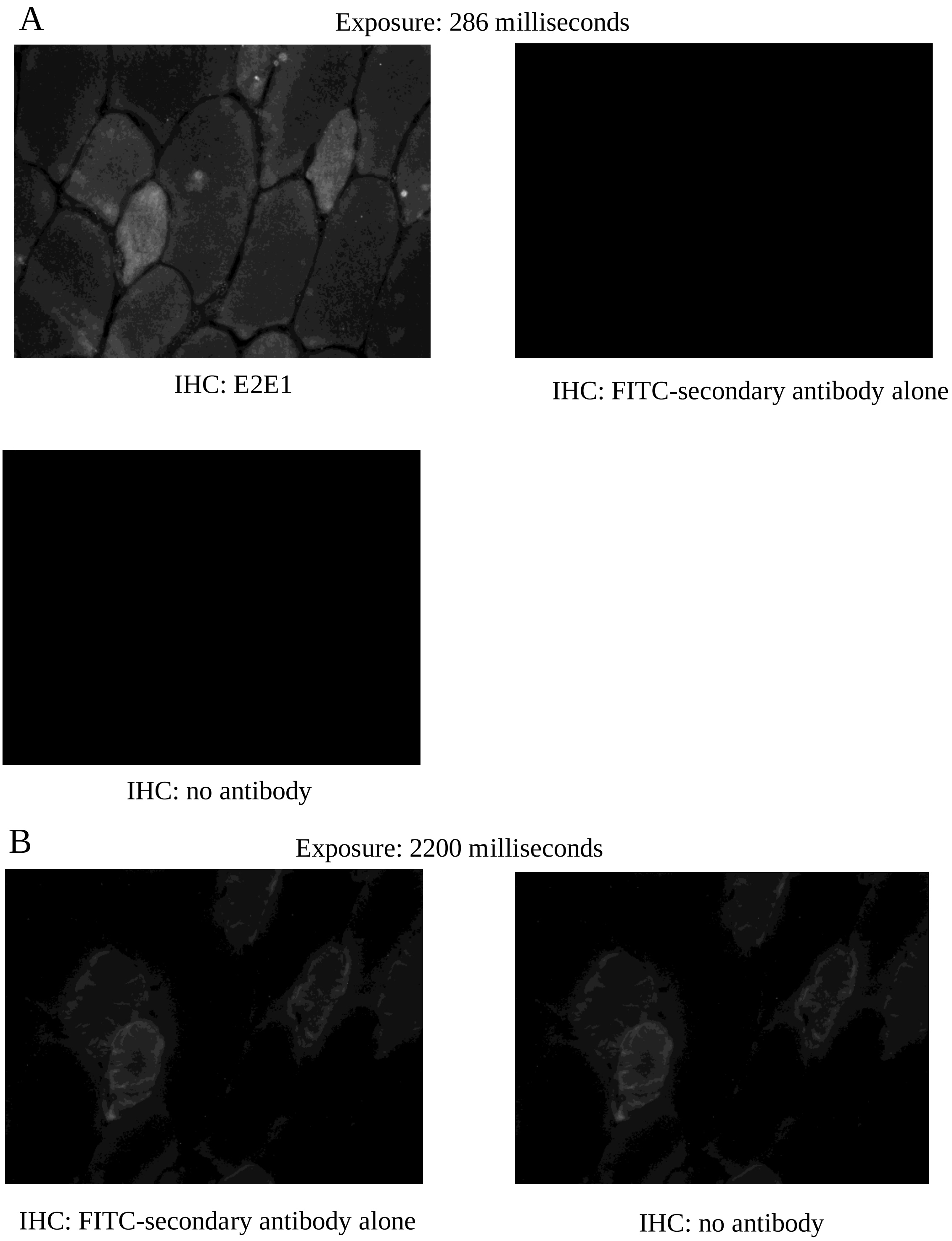

Supplemental Figure 1: Anti-E2E1 specifically detects E2E1 independently of autofluorescence in slow-twitch muscle fibers

IHC was performed as described in Figure 1 (upper left panel), with only the secondary FITC-conjugated antibody (upper right panel) or without any antibody (lower panel). Signal was acquired at 286 ms and the presence of the E2E1 antibody was necessary for detecting positive cells.

Exposure time was increased to 2200 ms for negative controls, which generated autofluorescence due to the presence of high concentrations of myoglobin in slow-twitch fibers. Please note that autofluorescence was mainly located close to the cytoplasmic membrane area while E2E1 was homogeneously distributed in the cell (compare with A, upper left panel).

Supplemental Figure 2

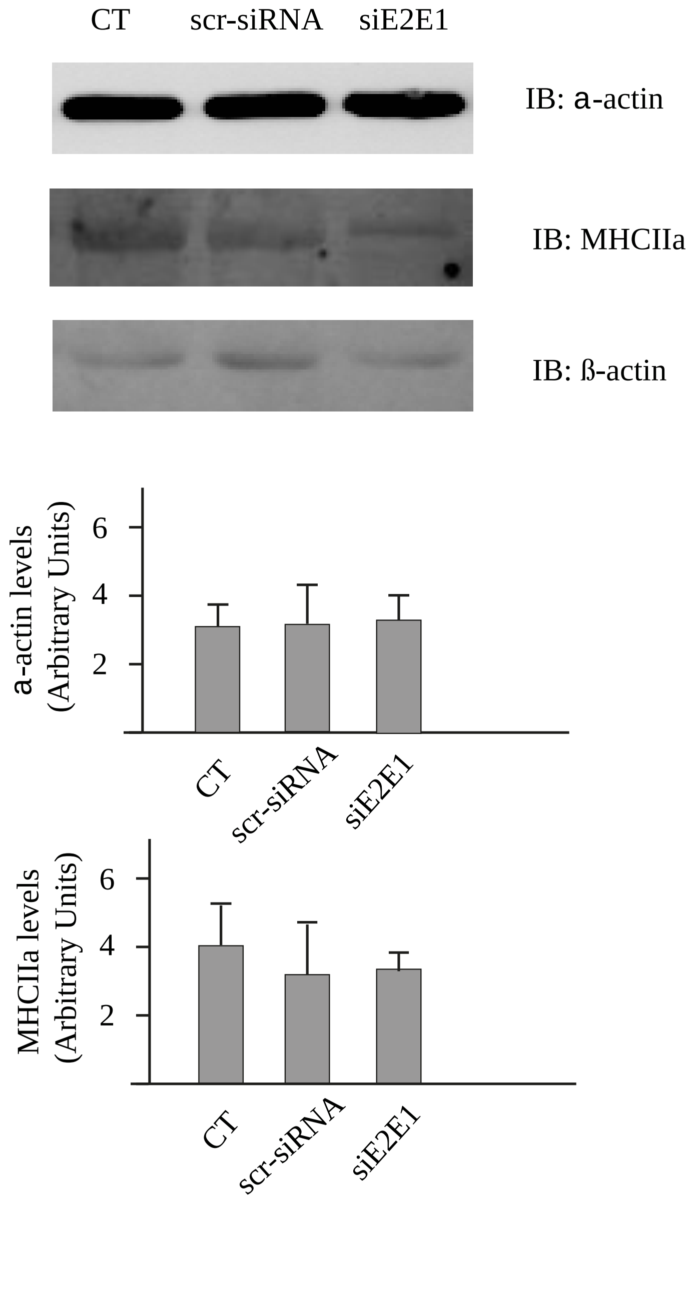

Supplemental Figure 2: T. anterior muscle were homogenized and the myofibrillar-enriched and soluble proteins were separated as described in the Materials and Methods section. Myofibrillar proteins were assayed for α-actin and MHCIIa levels by immunoblotting. ß-actin was used as a loading control. Values are means ± SE for n = 6 per group. E2E1 knockdown did not modify α-actin and MHCIIa levels, indicating that muscle atrophy was homogeneous.

Supplemental Table 1: List of shRNAs used for C2C12 myotubes knockdown experiments

**Genes**	**Sequence**
**MuRF1-shRNA1**	CCGGCGTGACCACAGAGGGTAAAGACTCGAGTCTTTACCCTCTGTGGTCACGTTTTTG
**MuRF1-shRNA2**	CCGGAGGAGGAGGAGTTTACAGAAGCTCGAGCTTCTGTAAACTCCTCCTCCTTTTTG
**MuRF1-shRNA3**	CCGGCATTGACTTTGGGACAGATGACTCGAGTCATCTGTCCCAAAGTCAATGTTTTTG
**UbE2E1-shRNA1**	CCGGGAGTTTGTTACCTCGTATATGCTCGAGCATATACGAGGTAACAAACTCTTTTTG
**UbE2E1-shRNA2**	CCGGCTATGAGTGGAGATCAACCATCTCGAGATGGTTGATCTCCACTCATAGTTTTTG

Supplemental Table 2: List of primers used for cloning into pcDNA™6.2-GW/EmGFP-miR vector and subsequent in vivo knock down experiments.

**Genes**	**sequence**
**UbE2E1-si1-top**	TGCTGAATGGTTGATCTCCACTCATAGTTTTGGCCACTGACTGACTATGAGTGGATCAACCATT
**UbE2E1-si1-bottom**	CCTGAATGGTTGATCCACTCATAGTCAGTCAGTGGCCAAAACTATGAGTGGAGATCAACCATTC
**UbE2E1-si2-top**	TGCTGTTCTCGGTCTGCTGGTTGGAGGTTTTGGCCACTGACTGACCTCCAACCAGACCGAGAA
**UbE2E1-si2-bottom**	CCTGTTCTCGGTCTGGGTTGGAGGTCAGTCAGTGGCCAAAACCTCCAACCAGCAGACCGAGAAC

Supplemental Table 3: List of primers used for qRT-PCR

Genes	Sequences
**MAFbx**	5' – AGTGAGGACCGGCTACTGTG -3'<br>5' - GATCAAACGCTTGCGAATCT -3'
**MuRF1**	5'- ATGGAGAACCTGGAGAAGCA -3'<br>5'- AACGACCTCCAGACATGGAC -3'
**YWHAZ**	5' – CTGGCCCTCAACTTCTCTGT – 3'<br>5' –AATGGCTTCATCGAAAGCTG – 3'
**Ppia**	5' – ACGCCACTGTCGCTTTTC – 3'<br>5' – CTGCAAACAGCTCGAAGGA – 3'
**36B4**	5' – TCACTGTGCCAGCTCAGAAC – 3'<br>5' – AATTTCAATGGTGCCTCTGG – 3'
